# Serum FGF21 levels are altered by various factors including lifestyle behaviors in male subjects

**DOI:** 10.1038/s41598-021-02075-8

**Published:** 2021-11-19

**Authors:** Kaori Nakanishi, Chisaki Ishibashi, Seiko Ide, Ryohei Yamamoto, Makoto Nishida, Izumi Nagatomo, Toshiki Moriyama, Keiko Yamauchi-Takihara

**Affiliations:** Health Care Division, Health and Counseling Center, Osaka University, 1-17 Machikaneyama, Toyonaka, Osaka 560-0043 Japan

**Keywords:** Biomarkers, Endocrinology, Health care, Risk factors

## Abstract

Fibroblast growth factor (FGF) 21 has various functions, including glucose and lipid metabolism. This cross-sectional study aimed to investigate specific conditions that might influence the functions of FGF21. 398 men who underwent a health examination were enrolled in this study. Physical and biochemical parameters and information on several lifestyle behaviors were obtained from all subjects. FGF21 levels correlated with age, body mass index (BMI), waist circumference (WC), systolic blood pressure (SBP), diastolic blood pressure (DBP), aspartate aminotransferase (AST), alanine aminotransferase (ALT), gamma-glutamyl transpeptidase (γ-GTP), uric acid, total cholesterol (TC), triglycerides (TG), high-density lipoprotein cholesterol (HDLC), fasting plasma glucose (FPG), and HbA1c. Moreover, FGF21 levels were significantly associated with lifestyle behaviors, including smoking status and breakfast and alcohol consumption frequency. Multivariable regression analysis showed that age, ALT, γ-GTP, smoking status, and breakfast and alcohol consumption frequency were independent variables for FGF21 levels. Assessment among the non-obese and obese groups showed that FGF21 levels correlated with WC, SBP, and TC only in the non-obese group. Thus, serum FGF21 levels were affected by several factors, including lifestyle behaviors, age, and liver function. To assess the functions of FGF21 in individuals, considering these factors would be essential.

## Introduction

Fibroblast growth factor (FGF) 21 is predominantly derived from the liver and is a member of the FGF19 subfamily, together with FGF15/19 and FGF23. Unlike other FGF subfamilies, members of the FGF19 subfamily exhibit hormone-like functions and require Klotho proteins: αKlotho and βKlotho, as co-factors to increase their binding affinity to FGF receptors^1^. FGF21 interacts with βKlotho and plays an important role in glucose and lipid metabolism. It regulates glucose uptake in adipocytes, enhances fatty acid oxidation in the liver, and inhibits lipogenesis^2,3^.

As FGF21 administration in obese animal models has shown an improvement in insulin sensitivity, a decrease in triglyceride and cholesterol levels, and a reduction in adiposity, FGF21 is expected as a new therapy for obesity and obesity-related diseases, including type 2 diabetes and nonalcoholic steatohepatitis^4–6^. Paradoxically, serum levels of FGF21 are increased in individuals with obesity, type 2 diabetes, and metabolic syndrome^7,8^. While this increase in FGF21 levels is regarded as a compensatory response or the FGF21-resistant state, the precise underlying mechanism for this increase remains unclear^9,10^.

We previously reported that FGF21 levels were high in smokers and negatively correlated with the metabolic syndrome-related cytokine, adiponectin. Interestingly, FGF21 levels were differentially associated with liver function and total cholesterol among smokers and never-smokers, suggesting that smoking stress affects the relationship between FGF21 and metabolic parameters^11^. Moreover, we demonstrated a sex-based difference in the relationship between FGF21 levels and metabolic parameters^12^. These results suggest that there may be some conditions that affect FGF21 levels.

Since FGF21 has various functions in multiple target organs, FGF21 biology is complicated and several uncertainties remain^13^. Therefore, in the present study, we focused on evaluating specific conditions that might influence the functions of FGF21 by assessing the associations of serum levels of FGF21 with physical parameters, biochemical parameters, and lifestyle behaviors using cross-sectional data of male subjects.

## Results

### Association of FGF21 levels with each parameter

The characteristics of the study participants are presented in Table 1. The median age and FGF21 levels were 42 (37–49) years and 165 (106–256) pg/mL, respectively.

**Table 1:** Characteristics of the study subjects.

n	398
Age (years)	42 (37–49)
BMI (kg/m^2^)	24.3 (21.9–9.3)
WC (cm)	86.0 (77.5–96.0)
SBP (mmHg)	124 (112–133)
DBP (mmHg)	78 (71–87)
AST (IU/l)	23 (18–32)
ALT (IU/l)	28 (17–47)
γ-GTP (IU/l)	39 (23–69)
Cr (mg/dl)	0.9 ± 0.1
UA (mg/dl)	6.3 ± 1.2
TC (mg/dl)	201 ± 32
TG (mg/dl)	105 (70–158)
HDLC (mg/dl)	52 (44–61)
FPG (mg/dl)	88 (83–94)
HbA1c (%)	5.3 (5.1–5.5)
FGF21 (pg/ml)	165 (106–256)

Data are expressed as means ± SD or medians (interquartile range).

BMI, body mass index; WC, waist circumference; SBP, systolic blood pressure; DBP, diastolic blood pressure; AST, aspartate aminotransferase; ALT, alanine aminotransferase; γ-GTP, gamma-glutamyl transpeptidase; Cr, creatinine; UA, uric acid; TC, total cholesterol; TG, triglycerides; HDLC, high-density lipoprotein cholesterol; FPG, fasting plasma glucose; FGF21, fibroblast growth factor 21.

Table 2 shows the correlations between serum levels of FGF21 and each physical and biochemical parameter. Serum levels of FGF21 showed significant positive correlations with age, Body mass index (BMI: body weight [kg] divided by squared height [m^2^]), waist circumference (WC), systolic blood pressure (SBP), diastolic blood pressure (DBP), aspartate aminotransferase (AST), alanine aminotransferase (ALT), gamma-glutamyl transpeptidase (γ-GTP), uric acid (UA), total cholesterol (TC), triglycerides (TG), fasting plasma glucose (FPG), and HbA1c and a negative correlation with high-density lipoprotein cholesterol (HDLC).

**Table 2:** Correlations of fibroblast growth factor 21 levels with physical and biochemical parameters.

	τ	P
Age	0.24*	< 0.0001
BMI	0.13*	0.0001
WC	0.15*	< 0.0001
SBP	0.20*	< 0.0001
DBP	0.21*	< 0.0001
AST	0.19*	< 0.0001
ALT	0.20*	< 0.0001
γ-GTP	0.31*	< 0.0001
Cr	− 0.02	0.571
UA	0.17*	< 0.0001
TC	0.10*	0.004
TG	0.23*	< 0.0001
HDLC	− 0.10*	0.003
FPG	0.17*	< 0.0001
HbA1c	0.13*	0.0001

*P < 0.005.

Abbreviations are as in Table 1.

### Smoking status, breakfast and alcohol consumption affect serum levels of FGF21

**Figure 1:**
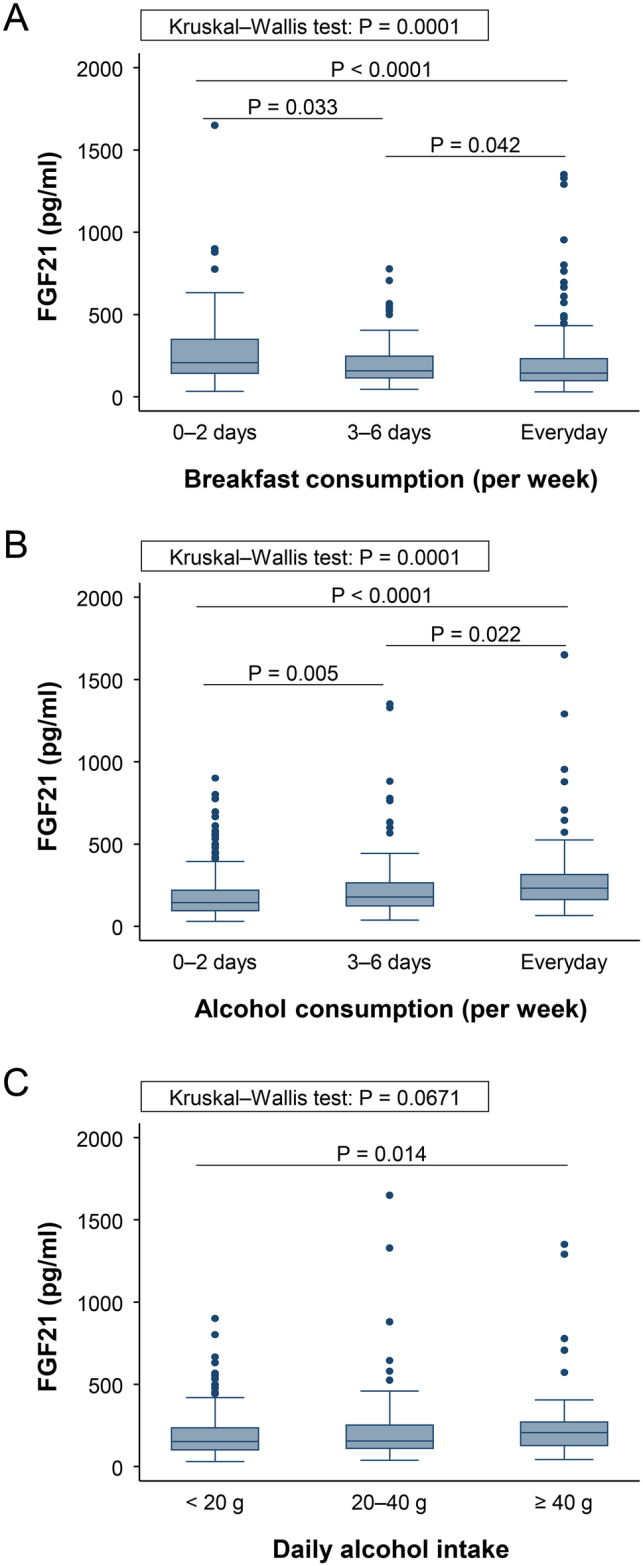
Serum levels of fibroblast growth factor (FGF) 21 are affected by breakfast and alcohol consumption. Associations of serum levels of FGF21 with (**A**) breakfast consumption frequency (0–2 days a week: n = 79, 3–6 days a week: n = 69, everyday: n = 240), (**B**) alcohol consumption frequency (0–2 days a week: n = 250, 3–6 days a week: n = 73, everyday: n = 75), and (**C**) daily alcohol intake (< 20 g: n = 170, 20–40 g: n = 78, ≥ 40 g: n = 42). Kruskal–Wallis test with Dunn’s post-hoc test was used to test between group differences. Data are shown as the sample minimum, lower quartile, median, upper quartile, and sample maximum. Dots represent outliers.

As we previously reported that serum levels of FGF21 were upregulated in smokers^11,12^, FGF21 levels were significantly higher in smokers [210 (124–353) pg/mL] than in never smokers [147 (101–224) pg/mL, p < 0.0001] and positively correlated with smoking status (τ = 0.14, p < 0.0001). Moreover, serum levels of FGF21 significantly negatively correlated with breakfast consumption frequency (τ =  − 0.12, p < 0.0001), and positively correlated with alcohol consumption frequency (τ = 0.15, p < 0.0001) and daily alcohol intake (τ = 0.08, p = 0.0237). We further assessed the associations of FGF21 levels with breakfast and alcohol consumption, as shown in Fig. 1. Serum levels of FGF21 were significantly increased in subjects whose breakfast consumption frequency was low (Fig. 1A). In addition, FGF21 levels were significantly increased in subjects whose alcohol consumption frequency was high (Fig. 1B) and tended to increase in subjects whose daily alcohol intake was high (Fig. 1C). These results suggest that lifestyle behaviors, including breakfast consumption and alcohol consumption affected the serum levels of FGF21 as smoking status.

### Age strongly associates with serum levels of FGF21

Multivariable regression analysis showed that age, ALT, γ-GTP, smoking status, and breakfast and alcohol consumption frequency were independent variables for FGF21 levels (Table 3). As correlation and regression analyses showed that age is a strong variable for FGF21 levels, we assessed FGF21 levels in each age group. The median serum levels of FGF21 in participants of age 30–39 years (n = 129), 40–49 years (n = 175), and ≥ 50 years (n = 94) were 119 (81–201) pg/mL, 181 (117–271) pg/mL, 214 (150–359) pg/mL, respectively. Significant differences were observed among age groups (p = 0.0001).

**Table 3:** Multivariable regression analysis of log-transformed fibroblast growth factor 21.

	β	P
Age	0.0108*	< 0.0001
WC	0.0008	0.634
DBP	0.0011	0.456
ALT	0.0011*	0.042
γ-GTP	0.0006*	0.049
UA	0.0144	0.256
TC	0.0001	0.829
TG	0.0002	0.234
HDLC	− 0.0013	0.339
FPG	0.0012	0.378
HbA1c	− 0.0326	0.415
Smoking status	0.0678*	0.022
Breakfast consumption frequency	− 0.0585*	0.001
Alcohol consumption frequency	0.0795*	< 0.0001

*P < 0.05.

Abbreviations are as in Table 1.

### Relationship between FGF21 and each parameter in non-obese and obese group

FGF21 is considered a potent inhibitor of obesity and FGF21 therapy has been identified as a possible treatment for obesity and obesity-related diseases in recent years^5^. Since FGF21 show a strong association with obesity, we categorized the study participants into non-obese (BMI < 25 kg/m^2^) and obese (BMI ≥ 25 kg/m^2^) groups and assessed the association between FGF21 levels and each parameter in both groups (Supplementary Table S1 online and Table 4).

**Table 4:** Correlations of fibroblast growth factor 21 with physical and biochemical parameters in the non-obese and obese groups.

	Non-obese		Obese	
	BMI < 25 kg/m^2^		BMI ≥ 25 kg/m^2^	
	τ	P	τ	P
Age	0.27*	< 0.0001	0.17*	0.001
BMI	0.09	0.058	− 0.04	0.486
WC	0.14*	0.002	0.00	0.965
SBP	0.21*	< 0.0001	0.05	0.298
DBP	0.21*	< 0.0001	0.12*	0.015
AST	0.16*	0.0004	0.13*	0.011
ALT	0.16*	0.0004	0.12*	0.022
γ-GTP	0.31*	< 0.0001	0.25*	< 0.0001
Cr	− 0.04	0.379	0.01	0.911
UA	0.12*	0.011	0.12*	0.018
TC	0.09*	0.040	0.07	0.182
TG	0.14*	0.002	0.24*	< 0.0001
HDLC	− 0.02	0.593	− 0.04	0.388
FPG	0.11*	0.014	0.16*	0.001
HbA1c	0.04	0.349	0.13*	0.012

*P < 0.05.

Abbreviations are as in Table 1.

Serum levels of FGF21 showed significant positive correlations with WC, SBP, and TC only in the non-obese group, while the positive correlation between FGF21 and HbA1c was observed only in the obese group. Thus, it can be suggested that FGF21 effects may differ depending on the BMI of each subject.

## Discussion

FGF21 has multiple functions, and recent studies have shown its various protective effects. Our previous study showed that FGF21 levels correlated with adiponectin, a metabolic syndrome-related cytokine that also has protective effects, including anti-inflammatory, anti-atherogenic, and anti-diabetic effects^11,14^. Since FGF21 regulates oxidative stress and attenuates inflammation, it is considered a stress-response factor^15,16^. In the present study, we analyzed the association of FGF21 levels with metabolic parameters and several lifestyle behaviors. We previously reported that smoking status affects the serum levels of FGF21^11^. In this study, we demonstrated that not only smoking, but also breakfast and alcohol consumption frequency affected the FGF21 levels. FGF21 has been reported as one of the genetic variants for breakfast skipping^17^, suggesting the strong association between breakfast consumption frequency and FGF21. Breakfast is considered the most important meal of the day and affects physical and mental health^18,19^. It is reported that breakfast skipping is associated with inflammation, related to an increase in WC and low-density lipoprotein cholesterol, and cardiovascular disease risk^20–23^. We found FGF21 levels to be increased in subjects with a low breakfast consumption frequency. Moreover, increased FGF21 levels were shown in subject with a high alcohol consumption frequency. FGF21 shows a protective effect on the liver damage induced by alcohol. In animal models, FGF21 administration reduces alcohol intake via the central nervous system interacting with βKlotho^15,24^. An increase in FGF21 levels in a stressed condition, including smoking, breakfast skipping, and alcohol ingestion, is suggested to be a protective response of FGF21.

Serum levels of FGF21 is known to have a large variation even in healthy population^25^. As presented in this study, the FGF21 levels might have been affected by some unknown factors, including lifestyle behaviors of each individual. Since FGF21 is considered a stress-response factor, it is quite possible that increased FGF21 levels are implicating the stressed condition. To our knowledge, there are no prior studies that address the influence of lifestyle behaviors on FGF21 levels, further studies are definitively needed to clarify these relationships.

While serum levels of FGF21 were related to many parameters, we found that age was a strong factor affecting FGF21 levels. Evaluation of serum levels of FGF21 in each age group showed that FGF21 levels were significantly increased along with aging. Aging is associated with various diseases, including cardiovascular diseases, cancer, and pulmonary diseases, which are known to be the leading causes of death worldwide^26^. Moreover, obesity and obesity-related diseases are associated with aging^27^. Previous studies have reported that FGF21 extended the lifespan of mice and showed a protective effect on age-related changes^28,29^. Since FGF21 can ameliorate both aging and metabolism, FGF21 is regarded as a key factor linking aging and metabolism^30^. In the present study, although the subjects were individuals who did not present any signs of disease, FGF21 levels were upregulated in the aged groups. Considering the anti-aging effects of FGF21, these increased FGF21 levels might be a compensatory response toward progress in aging and age-related changes. Since the age range was limited to median age 42 (37–49) years in the present study, we are willing to assess aged population in our further studies to elucidate this anti-aging effects of FGF21.

Individuals with obesity, in whom protective effects are not shown with increased serum levels of FGF21, are considered FGF21-resistant^7,9^. To evaluate differential functions of FGF21 between non-obese and obese subjects, we assessed the association of FGF21 with metabolic parameters among both groups. As previously reported, FGF21 levels were significantly high in the obese group. Serum levels of FGF21 correlated with more variables in the non-obese group than in the obese group. Moreover, WC, SBP, and TC correlated with FGF21 only in the non-obese group. WC is a known parameter for the definition of metabolic syndrome and is strongly associated with visceral fat accumulation^31^. Although both WC and FGF21 levels were high in obese subjects, there was no correlation between WC and FGF21. This disrupted relationship between FGF21 and WC suggests that the protective effects of FGF21 are attenuated in obese subjects. We previously reported that serum levels of FGF21 correlated with metabolic parameters differently among smokers and never-smokers^11^. We also confirmed that there were sex differences in the relationship between FGF21 levels and metabolic parameters^12^. These results show a variable relationship between FGF21 levels and metabolic parameters and that the effect of FGF21 may vary depending on the individual background. Since FGF21 is expected as a new therapy for obesity and obesity-related diseases^4,5^, effective conditions are needed to be evaluated.

FGF21 levels have been reported to be altered in various diseases^7,8,32^. However, as demonstrated in the present study, the serum levels of FGF21 are affected by several factors. Therefore, to assess the functions of FGF21 in subjects with such diseases, considering these factors and matching the conditions among the subjects is essential. Furthermore, as the mechanisms of change in FGF21 levels are still unclear, we believe that our findings might help elucidate the complicated biology of FGF21.

This study has some limitations. Since the study was exploratory research to find the FGF21 levels affecting factors, the sample size was not calculated in advance. We performed the post-hoc power analyses of our results: associations of FGF21 levels with breakfast consumption and alcohol consumption (Fig. 1) and multiple regression analysis (Table 3), and confirmed that the sample size in the study was adequate. Another limitation is that the study was a single center study with limited age range of subjects. Multicenter study with broad age range of subjects is preferable in the future study.

In conclusion, we evaluated the parameters and lifestyle behaviors that may influence FGF21 functions in the present study. Serum levels of FGF21 were affected by several lifestyle behaviors, including smoking status, breakfast consumption, and alcohol consumption. Moreover, in the FGF21 relating parameters, age was strongly associated with FGF21 levels. Serum levels of FGF21 are associated with metabolic parameters differently in non-obese and obese subjects.

## Methods

### Study subjects

This study included cross-sectional data obtained from employees at Osaka University. The subjects were randomly selected individuals who underwent an annual health examination at the Osaka University Health and Counseling Center. The inclusion and exclusion criteria for this study were as follows: (1) male subjects, (2) with no underlying diseases (3) had not taken any chronic or frequent medication for at least 1 year before their health examination, and (4) had not experienced any acute illness within the previous 2 weeks. A total of 398 Japanese men were enrolled in the study. This study was carried out in accordance with the Declaration of Helsinki and the Ethics Guidelines for Clinical Research from the Ministry of Health, Labour and Welfare and the Ministry of Education, Culture, Sports, Science and Technology. All experimental protocols in this study were approved by the Ethics Committee of Health and Counseling Center, Osaka University, and written informed consent was obtained from all subjects prior to participation in the study.

### Physical and biochemical parameters

BMI, WC at the umbilical level, SBP, DBP were measured as physical parameters.

Serum was collected from subjects between 9 and 11 A.M. after an overnight fast and stored at ≤  − 20 °C until assayed. Serum concentrations of AST, ALT, γ-GTP, creatinine, UA, TC, TG, HDLC, FPG, HbA1c, and FGF21 were measured as biochemical parameters from same blood sample. Serum levels of FGF21 were measured in duplicate for each sample using a commercially available sandwich enzyme-linked immunoassay system (DF2100; R&D Systems Inc., Minneapolis, USA) with assay range: 31.3–2000 pg/ml, intra-assay CV: 2.9–3.9%, and inter-assay CV: 5.2–10.9%. The methodology was performed according to the manufacturer’s instructions^11,15,33^.

### Lifestyle assessments

Information on the medical history, current treatments, smoking status, and lifestyle behaviors of the study participants were obtained via questionnaires. Each information was reconfirmed through expert interviews by trained nurses. Smoking status was semi-quantified as 0 = never smoker and 1 = smoker. Lifestyle behaviors, including breakfast and alcohol consumption frequency, and daily alcohol intake, were asked as follows, and each answer was semi-quantified using the following scales. Breakfast consumption frequency: “How many days a week do you eat breakfast?” on three scales: 1 = 0–2 days a week, 2 = 3–6 days a week, 3 = everyday; alcohol consumption frequency: “How many days a week do you drink alcohol?” on three scales: 1 = 0–2 days a week, 2 = 3–6 days a week, 3 = everyday; daily alcohol intake: “How many amounts of pure alcohol do you have on a typical day when you are drinking?” on three scales: 1 =  < 20 g, 2 = 20–40 g, 3 =  ≥ 40 g.

### Statistical analyses

All statistical analyses were performed using STATA 14 (STATA Corp LLC, College Station, TX, USA). The distribution of continuous variables was tested using the Shapiro–Wilk test. Normally distributed variables are presented as means ± standard deviation; non-normally distributed variables are reported as medians with interquartile ranges. Student’s t-test or the Mann–Whitney U test was used to compare differences between the two groups. Kendall’s rank correlation coefficient and multiple regression analysis were used to analyze the variables. For multi-group comparisons, the Kruskal–Wallis test with Dunn’s post-hoc test was used. Statistical significance was set at P < 0.05.

## Supplementary Information

Supplementary Table S1.

## Data Availability

The datasets generated during and/or analyzed during the current study are available from the corresponding author on reasonable request.
