# Supplementary material for: Serum FGF21 levels are altered by various factors including lifestyle behaviors in male subjects
Source: Sci Rep. 2021 Nov 19;11:22632. doi: 10.1038/s41598-021-02075-8 (PMC8604971; doi:10.1038/s41598-021-02075-8)
Supplement: Supplementary file 1 — Supplementary Table S1. [file 41598_2021_2075_MOESM1_ESM.docx]

**Supplementary Information**

**Serum FGF21 levels are altered by various factors
including lifestyle behaviors in male subjects**

Kaori Nakanishi, MD, PhD ^*^, Chisaki Ishibashi, MD, PhD, Seiko Ide, MD, PhD, Ryohei Yamamoto, MD, PhD, Makoto Nishida, MD, PhD, Izumi Nagatomo, MD, PhD, Toshiki Moriyama, MD, PhD, Keiko Yamauchi-Takihara, MD, PhD

**Supplementary Table S1. Characteristics of subjects in the non-obese and obese groups.**

|  | Non-obese |  | Obese | P |
| --- | --- | --- | --- | --- |
|  | BMI < 25 kg/m^2^ |  | BMI ≥ 25 kg/m^2^ |  |
| n | 219 |  | 179 | – |
| Age (years) | 41 (35–48) |  | 43 (39–49) * | 0.019 |
| BMI (kg/m^2^) | 22.2 (20.3–23.5) |  | 29.6 (27.9–31.1) * | < 0.0001 |
| WC (cm) | 79.0 (73.0–83.5) |  | 97.0 (93.0–102.0) * | < 0.0001 |
| SBP (mmHg) | 117 (108–128) |  | 130 (121–138) * | < 0.0001 |
| DBP (mmHg) | 73 (68–81) |  | 85 (77–91) * | < 0.0001 |
| AST (IU/l) | 19 (17–24) |  | 30 (24–38) * | < 0.0001 |
| ALT (IU/l) | 19 (14–27) |  | 47 (33–72) * | < 0.0001 |
| γ-GTP (IU/l) | 27 (19–48) |  | 58 (37–92) * | < 0.0001 |
| Cr (mg/dl) | 0.9 ± 0.1 |  | 0.9 ± 0.1 | 0.595 |
| UA (mg/dl) | 5.8 ± 1.2 |  | 6.8 ± 1.0* | < 0.0001 |
| TC (mg/dl) | 197 ± 30 |  | 207 ± 33* | 0.002 |
| TG (mg/dl) | 80 (58–116) |  | 142 (103–218) * | < 0.0001 |
| HDLC (mg/dl) | 58 (50–68) |  | 46 (40–52) * | < 0.0001 |
| FPG (mg/dl) | 86 (81–90) |  | 92 (86–101) * | < 0.0001 |
| HbA1c (%) | 5.2 (5.0–5.4) |  | 5.4 (5.2–5.8) * | < 0.0001 |
| FGF21 (pg/ml) | 145 (96–225) |  | 189 (124–300) * | 0.0001 |

* P < 0.05 versus non-obese group.

Data are expressed as means ± SD or medians (interquartile range).

BMI, body mass index; WC, waist circumference; SBP, systolic blood pressure; DBP, diastolic blood pressure; AST, aspartate aminotransferase; ALT, alanine aminotransferase; γ-GTP, gamma-glutamyl transpeptidase; Cr, creatinine; UA, uric acid; TC, total cholesterol; TG, triglycerides; HDLC, high-density lipoprotein cholesterol; FPG, fasting plasma glucose; FGF21, fibroblast growth factor 21.

As FGF21 relates strongly to obesity, we categorized the study participants into non-obese (BMI < 25 kg/m^2^) and obese (BMI ≥ 25 kg/m^2^) groups. The characteristics of each group are shown in Supplementary Table S1.
